# The long non-coding RNA MALAT1 is increased in renal ischemia-reperfusion injury and inhibits hypoxia-induced inflammation

**DOI:** 10.1080/0886022X.2018.1487863

**Published:** 2018-10-02

**Authors:** Hongyan Tian, Ming Wu, Peihui Zhou, Chuiguo Huang, Chaoyang Ye, Li Wang

**Affiliations:** a Department of Nephrology, The Ninth People’s Hospital, Shanghai Jiaotong University School of Medicine, Shanghai, China;; b Department of Nephrology, Shuguang Hospital Affiliated to Shanghai University of Traditional Chinese Medicine, Shanghai, China;; c Department of Urology, the second affiliated hospital of Zhengzhou University, Zhengzhou, Henan, China

**Keywords:** Acute kidney injury, ischemia/reperfusion, inflammation, long non-coding RNA, MALAT1

## Abstract

**Background:** To investigate the expression of long non-coding RNA metastasis-associated lung adenocarcinoma transcript 1 (MALAT1) in renal ischemia-reperfusion injury and explore its role in acute kidney injury.

**Methods:** 18 mice were randomly divided into a sham operation group (Sham) and an ischemia-reperfusion group (IR) in which animals were sacrificed at 6 h or 12 h after surgery. The kidneys were harvested to measure the expression of MALAT1 mRNA. HK2 cells were treated with cobalt chloride (CoCl_2_) to mimic hypoxia or transfected with siRNA to knockdown MALAT1 before CoCl_2_ treatment. After that, MALAT1 was analyzed by RT-PCR (reverse transcription-polymerase chain reaction). HIF-1ɑ (hypoxia-inducible factor-1 alpha) and NF-κB (nuclear factor-kappa B) was measured by Western blot. The concentrations of IL-6 (interleukin-6) and TNF-ɑ (tumor necrosis factor-alpha) in the media were detected by ELISA (enzyme-linked immunosorbent assay).

**Results:** The expression of MALAT1 in the IR (6 h/12 h) group was significantly higher than that in the sham group. In HK2 cells, MALAT1 was significantly increased at 1 h, 3 h, and 6 h after CoCl_2_ treatment but had reduced to the basal level at 12 h and 24 h. Knockdown of MALAT1 by siRNA significantly up-regulated the expression of HIF-1ɑ and NF-κB proteins in CoCl_2_-treated HK2 cells. In addition, the concentrations of IL-6 and TNF-ɑ were increased by MALAT1 siRNA transfection in CoCl_2_-treated HK2 cells.

**Conclusion:** The expression of MALAT1 is increased in renal ischemia-reperfusion injury. It is likely that MALAT1 inhibits the hypoxia-induced inflammatory response through the NF-κB pathway.

## Introduction

Acute kidney injury (AKI) is a common complication in hospitalized patients, especially in the intensive care unit (ICU). Approximately 30–60% of critically ill patients have AKI [1–3], while the incidence of AKI is approximately 21.6% in hospitalized adults [4]. The mortality due to AKI in the ICU can be as high as 60–70% [5,6], and in the hospital, approximately 20–40% of patients with AKI die with mortality being higher in patients with more severe AKI [7,8].

Ischemia-reperfusion injury (IRI) is one of the most common causes of acute kidney injury (AKI), which is associated with high rates of morbidity and mortality in clinics [9,10]. Inflammation is a complicated biological response that mediates tissue repair after injury, and it is also involved in the pathology of IRI-induced AKI, as shown in many experimental studies [11]. The transcriptional factor nuclear factor-kappa B (NF-κB) plays a central role in inflammation and it drives the transcription of a large array of pro-inflammatory cytokines such as tumor necrosis factor alpha (TNF-α), interleukin 6 (IL-6), and interleukin-1β (IL-1β) [10,12]. Animal studies have shown that inhibition of NF-κB diminishes renal inflammation and exerts renal benefits in AKI [10]. Hypoxia-inducible factor 1 (HIF-1α) is a central mediator of cellular adaptation to hypoxia and plays a pivotal role in the pathogenesis of AKI [13].Interestingly, the reciprocal regulation of HIF-1α and NF-κB has been shown, which further complicates our understanding of the underlining mechanism of AKI [14].

Long non-coding RNAs (lncRNAs) are non-protein-coding RNAs longer than 200 nucleotides in length. lncRNAs were formerly thought to be ‘Junk DNA’. However, recent studies have revealed that lncRNAs are important regulators in a variety of biological processes such as cell proliferation, apoptosis, and cell cycle progression [15]. MALAT1 (Metastasis-associated lung adenocarcinoma transcript 1) is one of the first identified lncRNAs, and it is widely and highly expressed in mammalian tissues, including kidneys [16]. Several studies have shown that MALAT1 is involved in diabetic nephropathy and promotes the inflammatory response upon high glucose stimulation [17]. A recent study showed that MALAT1 is strongly induced in mice organs exposed to hypoxia, with particularly high induction rates in spleen, kidney, testis, lung, and brain [16]. However, the expression and effects of MALAT1 in IRI-induced AKI remain unknown.

The aim of this study was to determine the expression and role of lncRNA MALAT1 in IRI-induced AKI.

## Materials and methods

### Animals and renal I/R injury model

Male C57BL/6 mice aged 8–10 weeks (18–22 g) were purchased from Shanghai Laboratory Animal Co., Ltd. (Shanghai, China) and housed at the Shanghai Institute of Materia Medica, Chinese Academy of Sciences, according to local regulations and guidelines. The mice model of renal I/R injury was established as described by Yan Jiang et al. [18]. Briefly, mice were anesthetized with intraperitoneal injections of pentobarbital (80 mg/kg), and a middle abdominal incision was made. Renal ischemia was achieved by clamping both renal pedicles for 40 min. Then, the clamps were removed to restore renal blood flow. Sham-operated mice also underwent the same surgical procedure without clamping of the bilateral renal pedicles. After 6 h or 12 h of recovery, mice (*n* = 6 per group) were sacrificed, and blood samples and renal tissues were harvested for further analysis. The experimental protocol of the current study was approved by the local ethics committee. Serum creatinine (Cr) and blood urea nitrogen (BUN) levels were measured by a routine protocol in the clinical laboratory of the Ninth People’s Hospital affiliated with Shanghai Jiaotong University.

### Cell culture

Human proximal tubular epithelial cells (HK2) were purchased from the cell bank of Chinese Academy of Sciences and were cultured in DMEM/F12 medium with 10% fetal bovine serum (Gibco, Carlsbad, CA), 100 U/ml penicillin (Gibco, Carlsbad, CA), and 100 µg/ml streptomycin (Gibco, Carlsbad, CA). To mimic hypoxia, HK2 cells at 80% confluence were treated with 200 µM CoCl2 (Sigma, St. Louis, MO) for subsequent evaluation of MALAT1 expression at 1 h, 3 h, 6 h, 12 h, and 24 h.

### Cell transfection

Chemically synthesized siRNAs (Invitrogen, Shanghai, China) were transfected into cultured HK2 cells with Lipofectamine 2000 (Invitrogen, Carlsbad, CA) for 6 h and then provided with new medium (DMEM/F12 + 10% FBS). Twenty-four hours after transfection, HK2 cells were treated with 200 µM CoCl2 in DMEM/F12 medium to mimic the effects of hypoxia for another 24 h. Cells were harvested to isolate total RNAs and protein. The siRNA sequences for human siMALAT1 were as follows: sense,5′-GATCCATAATCGGTTTCAAGG-3′; antisense, 5′-TTGAAACCGATTATGGATCAT-3′. The oligo sequences for human nonsense control siRNA were as follows: sense, 5′-UUCUCCGAACGUGUCACGUTT-3′; antisense, 5′-ACGUGACACGUUCGGAGAATT-3′. Real-time quantitative PCR RNA was extracted using TRizolTM reagent (15596018, Invitrogen, Carlsbad, CA). After extraction, 2 µg of total RNA was used to make cDNA using a reverse transcription kit (PrimeScript™ RT reagent Kit, Takara, RR037A, Mountain View, CA ). The levels of the target gene MALAT1 and housekeeping gene GAPDH were measured using a commercial kit (SYBR^®^ Premix Ex Taq™, Takara, RR420A, Mountain View, CA). The primer sequences for mice MALAT1 were as follows: forward primer,

5′-CACTTGTGGGGAGACCTTGT-3′; reverse primer, 5′-TGTGGCAAGAATCAAGCAAG-3′. The primer sequences for mice GAPDH were as follows: forward primer, 5′-CTCATGACCACAGTCCATGC-3′; reverse primer, 5′-CACATTGGGGGTAGGAACAC-3′. The primer sequences for human MALAT1 were as follows: forward, 5′-GTGATGCGAGTTGTTCTCCG-3′; reverse, 5′-CTGGCTGCCTCAATGCCTAC-3′. The primer sequences for human GAPDH were as follows: forward, 5-CCACCCATGGCAAATTCC-3′; reverse, 5′-TGGGATTTCCATTGATGACAAG-3′. The primers were synthesized by Invitrogen, Shanghai, China. The results were expressed as log10 (2^−ΔΔCt^).

### Western blot

Kidney tissues or HK2 cells were lysed with ice-cold lysis buffer supplemented with protease inhibitors (RIPA, Beyotime, P0013B). Proteins were resolved on 10% Tris-glycine gels and transferred to a nitrocellulose membrane. After blocking with skim milk, the membrane was incubated with the primary antibody at 4 °C overnight. Membranes were washed three times and then incubated with the peroxidase-conjugated secondary antibody. After washing the membranes three times, the specific protein bands were detected using enhanced chemiluminescence reagents. The primary antibodies for NF-κB (p65, ab16502) and GAPDH (ab8245) were purchased from Abcam (Cambridge, MA). The antibodies for NF-κB (p-p65, sc-372m) were from Santa Cruz Biotechnology (Santa Cruz, CA). The antibody for HIF-1α was purchased from BD (610958, Shanghai, China).

### ELISA assay

The supernatant from the HK2 culture was collected for the ELISA experiment. Human TNF-α and IL-6 kits were obtained from Jiancheng Company (H052, H007, Nan Jing, China). The concentrations of TNF-α and IL-6 were measured according to the instructions of the commercial kits.

### Statistical analysis

Statistical analyses were performed using the software GraphPad Prism 6.01. Data are expressed as the mean ± SD. Analysis of variance (ANOVA) was used to test for significant differences among the groups. A *p* value <.05 was considered as statistically significant.

## Results

### The expression of lncRNA MALAT1 was increased in the kidneys of mice after renal ischemia/reperfusion injury

**Figure 1.**
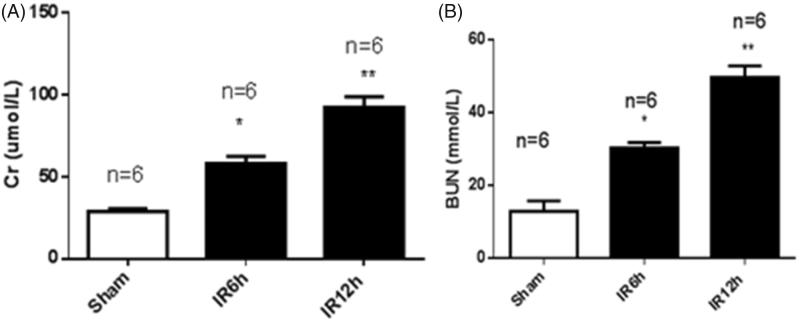
The renal function of mice with ischemia/reperfusion-induced acute kidney injury. Serum was collected at 6 h and 12 h after sham (*n* = 6) or renal ischemia/reperfusion injury (IRI) (6 h, *n* = 6; 12 h, *n* = 6) in mice. Blood urea nitrogen (BUN) (A) and serum creatinine (Scr) (B) were measured. *p < .05 vs the sham group; **p < .01 vs the sham group.

**Figure 2.**
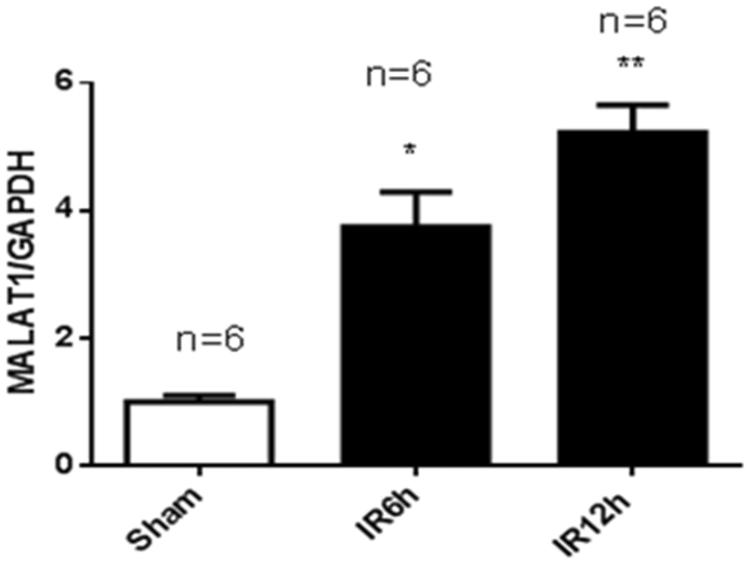
Renal ischemia/reperfusion injury increased the expression of MALAT1 in vivo. Kidneys were collected at 6 h and 12 h after sham (*n* = 6) or renal ischemia/reperfusion injury (IRI) (6 h, *n* = 6; 12 h, *n* = 6) in mice. The expression of MALAT1 was measured by RT-PCR, and the ratio of MALAT1/GAPDH was normalized to the sham group. *p < .05 vs the sham group; **p < .01 vs the sham group.

To validate the mouse model of renal ischemia/reperfusion injury (IRI), we measured the serum creatinine (Scr) and blood urea nitrogen (BUN) levels at 6 h and 12 h after IRI and at 6 h after sham operation. As shown in Figure 1, the Scr level was 28.9 ± 4.0 µmol/L in the sham group. In the IRI group, the Scr level was 50.2%greater at 6 h and 68.7% greater at 12 h than the Scr level of the sham-operated controls. The BUN level in the sham group was 11.9 ± 2.8 mmol/L. In the IRI group, the BUN level was 60.9% higher at 6 h and 76.1% higher at 12 h than that of the sham group (Figure 1). As shown in Figure 2, the expression of lncRNA MALAT1 in kidney tissues was 3.74-fold greater at 6 h and 5.22-fold greater at 12 h in the IRI group than that in the sham group.

### The expression of MALAT1 in HK2 cells was increased under CoCl2-induced hypoxia

**Figure 3.**
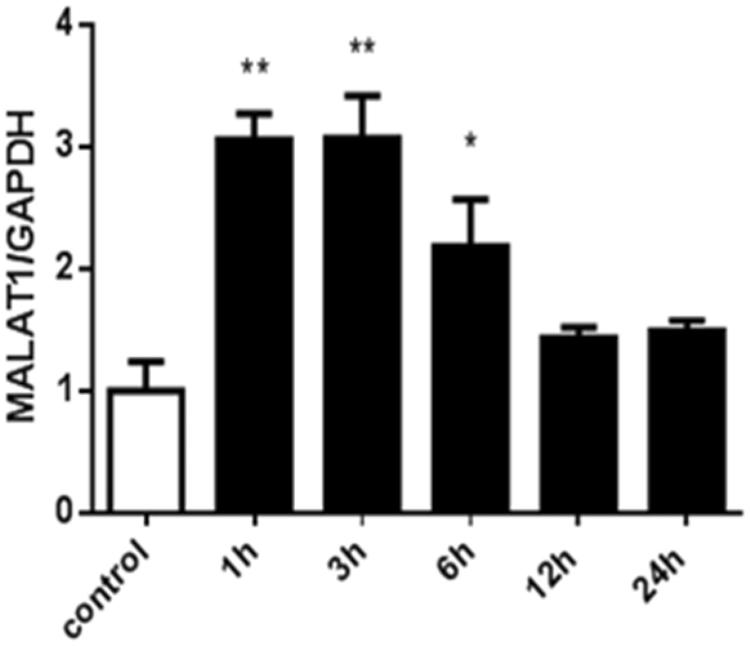
The expression of MALAT1 in HK2 cells at different time points after CoCl_2_ treatment. HK2 cells were treated with normal medium or 200 µmol/L of CoCl_2_ for various durations. The expression of MALAT1 was analyzed by RT-PCR, and the ratio of MALAT1/GAPDH was normalized to the control group. Data are representative of three independent experiments performed in triplicate. *p < .05 vs the control group; **p < .01 vs the control group.

To further confirm the regulation of MALAT1 expression by hypoxia, HK2 cells were treated with the chemical compound CoCl_2_. Figure 3 shows that CoCl_2_-induced hypoxia significantly increased the expression of MALAT1 in HK2 cells at 1 h, 3 h, and 6 h compared with the control level of expression. The expression of MALAT1 returned to the control level at 12 h and 24 h after CoCl_2_ treatment (Figure 3).

### Knockdown of MALAT1 expression promoted the hypoxia-induced inflammatory response in HK2 cells

**Figure 4.**
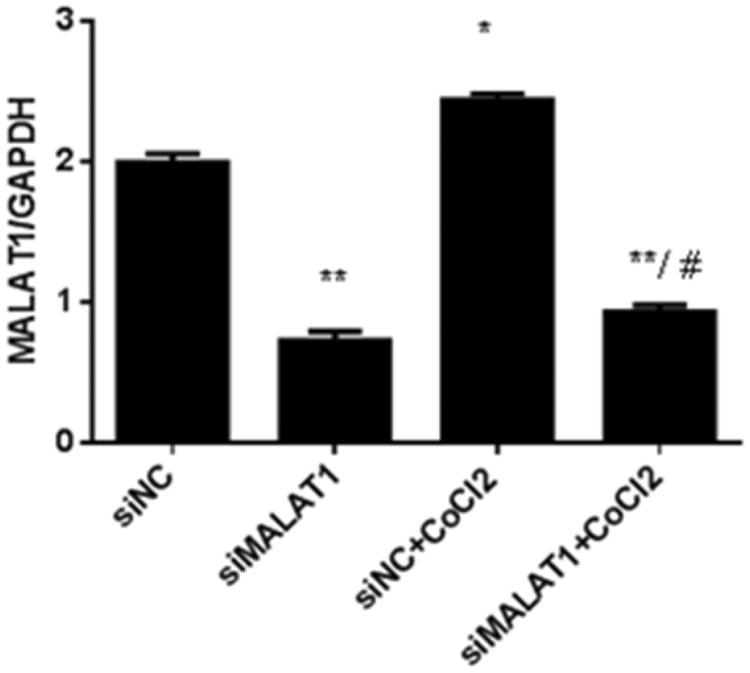
The effect of MALAT1 siRNA on MALAT1 expression in CoCl_2_-treated HK2 cells. HK2 cells were transfected with siRNA for MALAT1 (siMALAT1) or non-sense control siRNA as a control treatment (siNC). Twenty-four hours after transfection, HK2 cells were treated with normal media or 200 µmol/L CoCl_2_ for 3 h. The expression of MALAT1 was analyzed by RT-PCR, and the ratio of MALAT1/GAPDH was normalized to the control group. Data are representative of three independent experiments performed in triplicate. *p < .05 vs the siNC group; **p < .01 vs the siNC group; #*p* < .05 vs the siNC + CoCl_2_ group.

Real-time PCR analysis showed that 3 h of CoCl_2_ treatment significantly increased MALAT1 expression by 22% in the non-sense siRNA transfected (siNC) control HK2 cells (Figure 4). The expression of MALAT1 in HK2 cells was significantly decreased by 62% after 24 h MALAT1 siRNA transfection (siMALAT1) under CoCl_2_ treatment (Figure 4). We analyzed the expression of NF-κB (p-p65/p65) protein by Western blotting.

**Figure 5.**
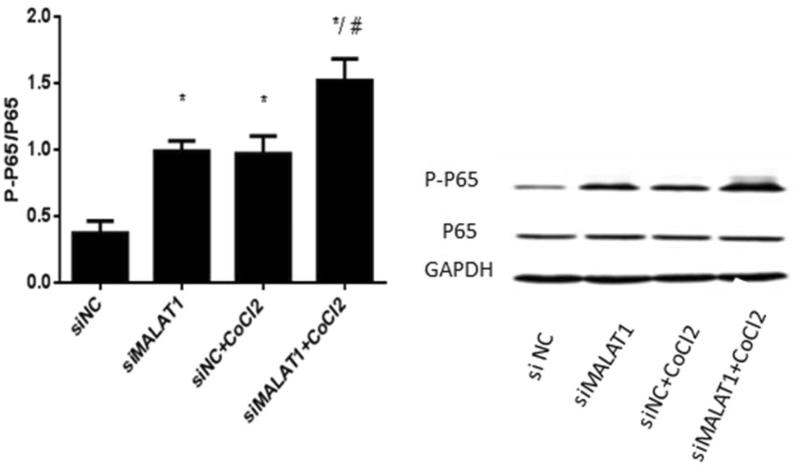
The expression of NF-κB (p-p65/p65) in HK2 cells under CoCl2-induced hypoxia. HK2 cells were transfected with siRNA for MALAT1 (siMALAT1) or non-sense control siRNA as a control treatment (siNC). Twenty-four hours after transfection, HK2 cells were treated with normal media or 200 µmol/L CoCl_2_ for 3 h. Phosphorylation and total P65 were analyzed by Western blot and then quantified by α-tubulin. Data are mean ± SD of three independent experiments. *p < .05 vs the siNC group; #*p* < .05 vs the siNC + CoCl_2_ group.

**Figure 6.**
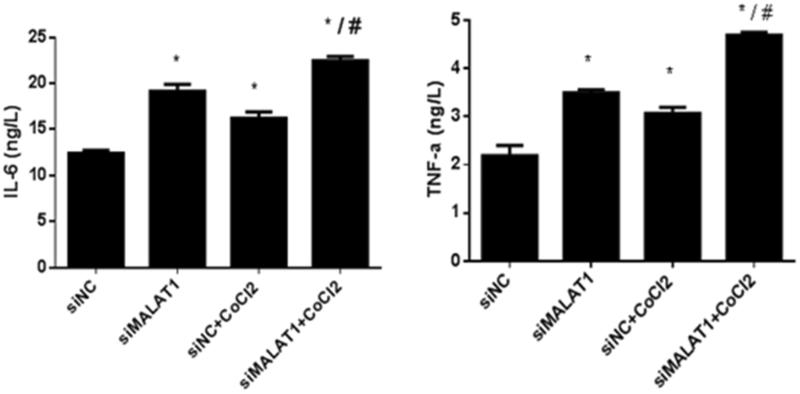
IL-6 and TNF-α secretion in HK2 cells under CoCl_2_-induced hypoxia. HK2 cells were transfected with siRNA for MALAT1 (siMALAT1) or non-sense control siRNA as a control treatment (siNC). Twenty-four hours after transfection, HK2 cells were treated with normal media or 200 µmol/L CoCl_2_ for 3 h. The concentrations of IL-6 and TNF-α in the media were measured by ELISA. Data are representative of three independent experiments performed in triplicate. *p < .05 vs the siNC group; #*p* < .05 vs the siNC + CoCl_2_ group.

Figure 5 shows that the expression of NF-κB (p-p65/p65) protein was significantly increased upon CoCl_2_ treatment in the siNC group and that the transfection of MALAT1 siRNA significantly increased the expression of NF-κB (p-p65/p65) protein regardless of the presence of CoCl_2_. The secretion of two inflammatory cytokines (IL-6 and TNF-α) was measured by ELISA. The concentrations of IL-6 and TNF-α in the media of CoCl_2_ treated cells were 31% and 40% greater, respectively, than those in the media of the siNC control cells (Figure 6). The transfection of MALAT1 siRNA increased the concentrations of IL-6 and TNF-α in CoCl_2_-treated HK2 cells by 38% and 53%, respectively, compared with concentrations in the siNC + CoCl_2_ group (Figure 6).

### MALAT1 siRNA increased the expression of HIF-1α protein in HK2 cells under CoCl_2_ treatment

**Figure 7.**
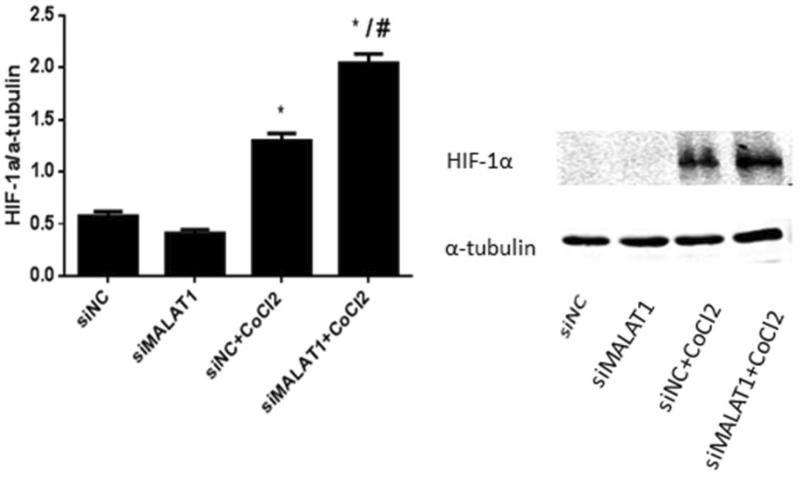
The expression of HIF-1α in HK2 cells after CoCl_2_ treatment and MALAT1 siRNA transfection. HK2 cells were transfected with siRNA for MALAT1 (siMALAT1) or non-sense control siRNA as a control treatment (siNC). Twenty-four hours after transfection, HK2 cells were treated with normal media or 200 µmol/L CoCl_2_ for 3 h. The expression of HIF-1α was analyzed by Western blot and quantified by α-tubulin. Data are mean ± SD of three independent experiments. *p < .05 vs the siNC group; #*p* < .05 vs the siNC + CoCl_2_ group.

The expression of HIF-1α protein was very low in the siNC control and siMALAT1 control groups (Figure 7). Treatment with CoCl_2_ induced HIF-1α expression in non-sense siRNA transfected HK2 cells, and the expression of HIF-1α was further enhanced by MALAT1 siRNA transfection (Figure 7).

## Discussion

In this study, we demonstrated that (1) the expression of the lncRNA MALAT1 was up-regulated in a mouse model of AKI induced by ischemia/reperfusion and (2) knocking-down MALAT1 enhanced HK2 cell inflammation (IL-6/TNF-α) in chemically mimicked hypoxia. The mechanism likely involves the NF-κB pathway, which was over-activated after knocking-down the expression of MALAT1 in CoCl_2_-treated HK2 cells.

MALAT1 is a long non-coding RNA, which is highly conserved and exists broadly in many mammalian organs. Lelli et al. found that the expression of MALAT1 was increased under hypoxic conditions in several cancer cell lines and mouse kidney epithelial cells [16]. However, it remained unknown whether the expression of MALAT1 is increased in renal ischemia/reperfusion injury. In the current study, we observed that MALAT1 was significantly increased in a mouse model of renal ischemia/reperfusion injury. Moreover, we found that with CoCl_2_ treatment, the expression of MALAT1 was up-regulated in HK2 human kidney epithelial cells. These results suggest that the lncRNA MALAT1 might play an important role in AKI. A severe inflammatory response is the typical pathological characteristic of ischemia reperfusion-induced acute renal failure, as has been shown in many animal and human studies [15,17]. In renal ischemia reperfusion injury, the transcription factor nuclear factor κB (NF-κB) is activated, which in turn activates the expression of many inflammatory factors such as IL-6 and TNF-α and promotes inflammatory cell infiltration, cell apoptosis, and tissue damage [19]. Recent studies have indicated that MALAT1 may play a vital role in the inflammation response. Zhao et al. found that MALAT1 was up-regulated in lipopolysaccharide (LPS)-activated macrophages and that knockdown of MALAT1 increased LPS-induced TNF-α and IL-6 expression. Mechanistically, MALAT1 was found to interact with NF-κB in the nucleus and inhibit its DNA-binding activity, consequently decreasing the production of inflammatory cytokines [16]. Therefore, we hypothesized that MALAT1 might play an important role in the process of inflammation in AKI. Our data showed that knockdown of MALAT1 in HK2 cells activated NF-κB (p-p65/p65) under chemically mimicked hypoxia and promoted the secretion of inflammatory cytokines, suggesting an anti-inflammatory role of MALAT1 in AKI.

The transcriptional factor hypoxia-inducible factor-1 (HIF-1) is a central player in hypoxia and consists of an oxygen regulated α-subunit and a constitutive β-subunit. In normoxia, hydroxylation leads to the ubiquitination of the HIF-1α proteins and inhibits its activity [20]. Under hypoxia, HIF-1α is activated and promotes the transcription of downstream hypoxia responsive genes (HRGs) that are involved in multiple cellular processes such as energy metabolism, angiogenesis, proliferation and metastasis [21–25]. Lelli et al. showed that knockdown of HIF-1α by transfecting shHIF-1α in hypoxic MCF-7 breast cancer cells increased the expression of MALAT1 [14]. Conversely, we found that knockdown of MALAT1 up-regulated the expression of HIF-1α in CoCl_2_-treated cells, suggesting that a mutual inhibitory mechanism may exist between MALAT1 and HIF-1α. Jiang et al. [14] showed that under hypoxic conditions, the NF-κB p50/p65 complex binds to the HIF-1α promoter and thereby increases its transcription [26]. Our *in vitro* study showed that knockdown of MALAT1 activates NF-κB and increases HIF-1α expression, suggesting that MALAT1 may regulate HIF-1α expression through NF-κB under chemically mimicked hypoxia.

Taken together, our results show that the expression of MALAT is significantly increased in *in vitro* and *in vivo* AKI models. MALAT1 might inhibit the renal inflammatory reaction induced by IRI in the kidney. Further studies should be performed to confirm the protective role of MALAT1 in animal models of AKI.
